# Supplementary material for: Phonons as a platform for non-Abelian braiding and its manifestation in layered silicates
Source: Nat Commun. 2022 Jan 20;13:423. doi: 10.1038/s41467-022-28046-9 (PMC8776786; doi:10.1038/s41467-022-28046-9)
Supplement: Supplementary file 1 — Supplementary Information [file 41467_2022_28046_MOESM1_ESM.pdf]

Supplementary Information for "Phonons as a platform for non-Abelian braiding and its manifestation in layered silicates"

Bo Peng,[1,*] Adrien Bouhon,[2,†] Bartomeu Monserrat,[1,3,‡] and Robert-Jan Slager[1,§]

[1] *TCM Group, Cavendish Laboratory, University of Cambridge, J. J. Thomson Avenue, Cambridge CB3 0HE, United Kingdom*

[2] *Nordic Institute for Theoretical Physics (NORDITA), Stockholm, Sweden*

[3] *Department of Materials Science and Metallurgy, University of Cambridge, 27 Charles Babbage Road, Cambridge CB3 0FS, United Kingdom*

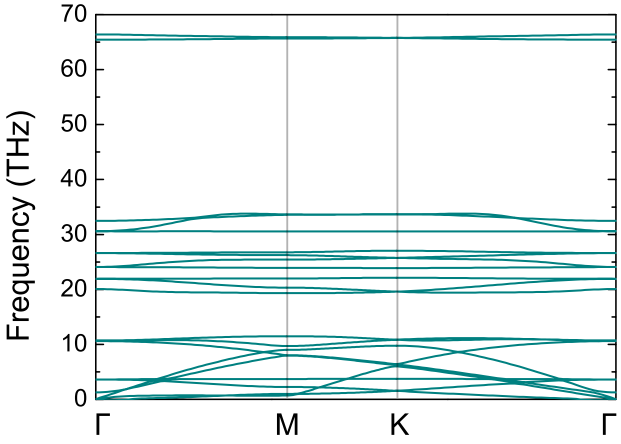

Supplementary Fig. 1. Full phonon dispersion of unstrained silicate without an electric field. The high-frequency bands are dominated by vibrations of the passivating hydrogen atoms, while the bands below 35 THz are dominated by vibrations of the silicon and oxygen atoms.

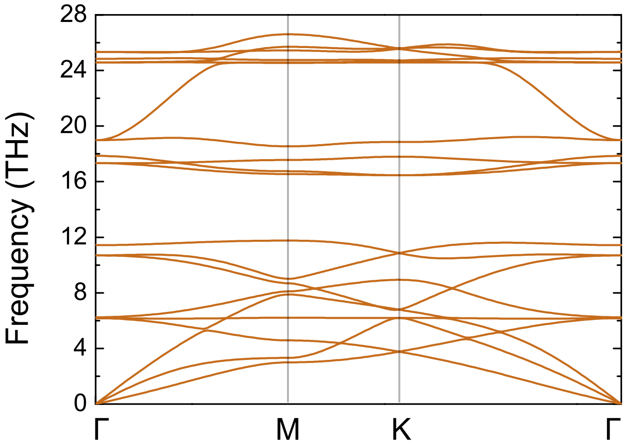

Supplementary Fig. 2. Phonon dispersion of 7% strained monolayer Si_2O_3 under an electric field of 2.0 eV/Å.

---

* bp432@cam.ac.uk

† adrien.bouhon@su.se

‡ bm418@cam.ac.uk

§ rjs269@cam.ac.uk

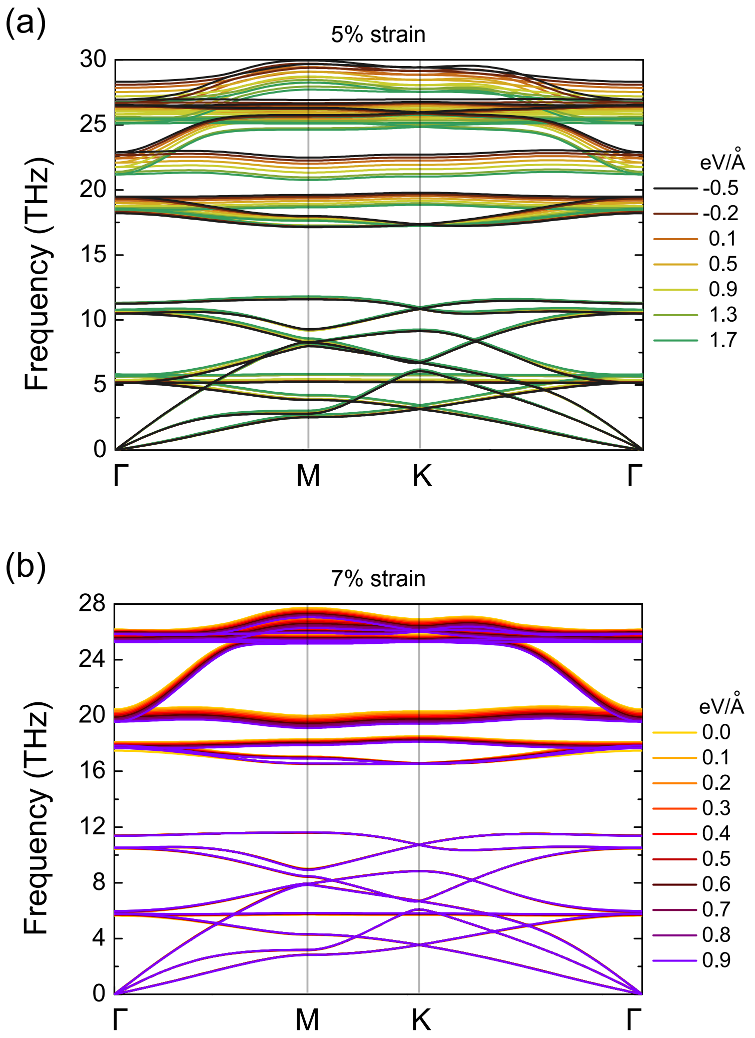

Supplementary Fig. 3. Phonon dispersion of (a) 5% and (b) 7% strained monolayer Si_2O_3 under different electric fields.

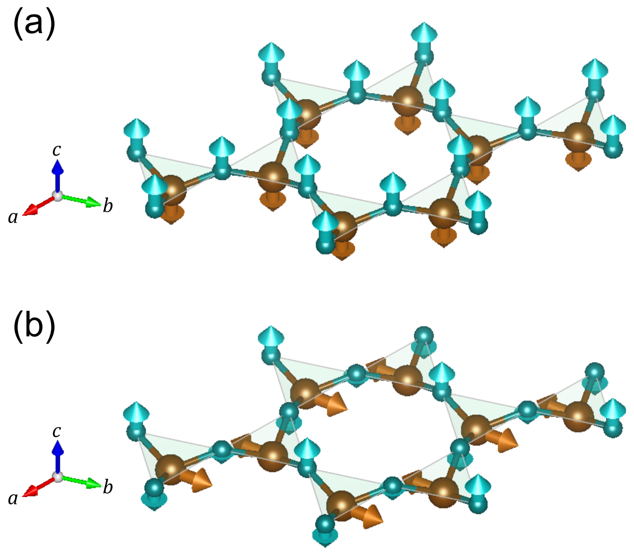

Supplementary Fig. 4. Phonon vibrational mode of (a) $\mathcal{B}_{10}$ and (b) $\mathcal{B}_{11,12}$ at Γ.

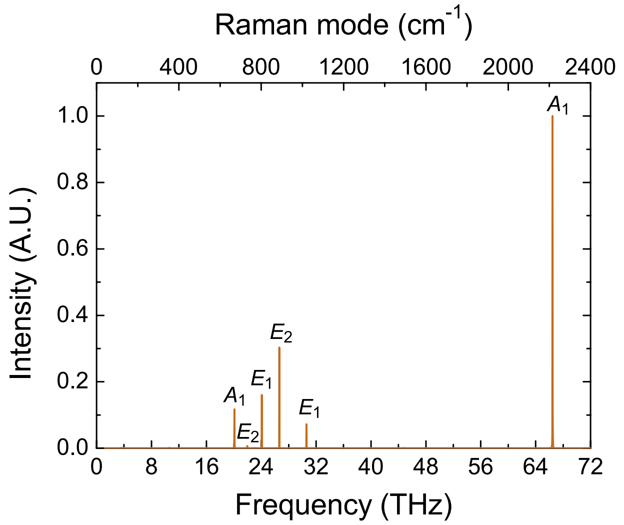

Supplementary Fig. 5. Simulated Raman spectrum (A.U. = arbitrary units) of unstrained monolayer silicate Si_2O_3 without an electric field. The lowest A_1 and E_2 modes around 18-20 THz belong to group 1, the E_1 and E_2 modes around 24-28 THz belong to group 2, and the E_1 mode around 32 THz belongs to group 3.

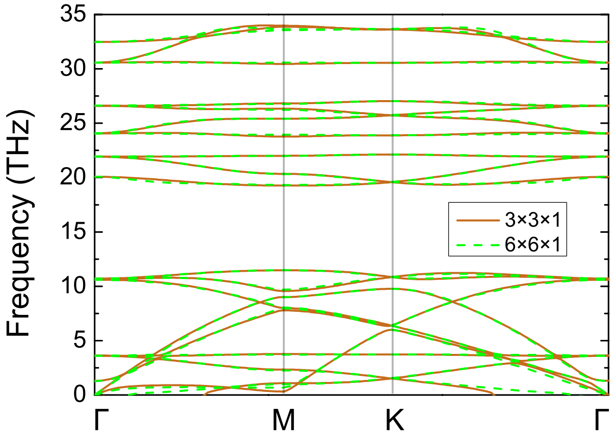

Supplementary Fig. 6. Phonon spectra calculated with a $3 \times 3 \times 1$ and a $6 \times 6 \times 1$ supercell.
